# Correction: A systematic review of exercise testing in patients with intermittent claudication: A focus on test standardisation and reporting quality in randomised controlled trials of exercise interventions

**DOI:** 10.1371/journal.pone.0315609

**Published:** 2024-12-06

**Authors:** Stefan T. Birkett, Amy E. Harwood, Edward Caldow, Saïd Ibeggazene, Lee Ingle, Sean Pymer

The phrase "Part 1—evaluating walking capacity" is missing from the title. The correct title is: A systematic review of exercise testing in patients with intermittent claudication: A focus on test standardisation and reporting quality in randomised controlled trials of exercise interventions: Part 1—evaluating walking capacity. The correct citation is: Birkett ST, Harwood AE, Caldow E, Ibeggazene S, Ingle L, Pymer S (2021) A systematic review of exercise testing in patients with intermittent claudication: A focus on test standardisation and reporting quality in randomised controlled trials of exercise interventions: Part 1—evaluating walking capacity. PLOS ONE 16(5): e0249277. https://doi.org/10.1371/journal.pone.0249277
