# Supplementary material for: A systematic review of exercise testing in patients with intermittent claudication: A focus on test standardisation and reporting quality in randomised controlled trials of exercise interventions
Source: PLoS One. 2021 May 3;16(5):e0249277. doi: 10.1371/journal.pone.0249277 (PMC8092776; doi:10.1371/journal.pone.0249277)
Supplement: S1 Table — (DOCX) [file pone.0249277.s002.docx]

S1 Table - Trial characteristics

| **Author** | **Year** | **Country** | **Exercise arm(s)** | **Comparator arm(s)** |
| --- | --- | --- | --- | --- |
| Allen | 2010 | USA | Treadmill walking SEP  HEP  SEP: F - 3 days per week for 12 weeks I - initial intensity set to workload that brought on pain in the baseline treadmill test and then walked until claudication pain became moderately severe in SEP. In subsequent visits the speed/elevation was increased once the subject could walk 8-10 min or longer without reaching mod pain.  T - 30-40 minutes of walking time T - treadmill walking  HEP:  F - 3 days per week for 12 weeks I - not stated  T - 30 minutes  T - walking  *asked to keep "careful notes" regarding activity and called every 3 weeks | Healthy controls |
| Baker | 2017 | USA | Treadmill walking SEP  SEP: F - 3 days per week for 12 weeks I - 2.0mph to mild / moderate pain. Allowed to rest until pain has abated and then resume walking  T - 60 minutes per session T - Walking on a treadmill | Usual care control group |
| Brenner | 2019 | Canada | HEP  Structured low intensity walking  F - 5 days per week for 12 weeks I - <40% of HRR and RPE of 11-13 until they felt minimal claudication pain on the Borg-CR 10-scale <2 T - NR but 0.4 km per day until reaching 3.2 km per day (gradual increase every 2 weeks) T - Walking | Usual care control group |
| Bronas | 2011 | USA | Arm ergometry SEP  Treadmill walking SEP  Arm-ergometry: F - 3 days per week for 12 weeks I - 13-15 on the BORG 20 RPE scale T - 60 minutes including 5 min warm up and cool down T - arm ergometry  Treadmill walking:  F - 3 days per week for 12 weeks I - 3/5 out of 0-5 claudication pain scale then stopped and sat down until pain subsided before resuming T - 60 minutes including 5 min warm up and cool down T - treadmill walking | Usual care control group |
| Bulinska | 2015 | Poland | Treadmill walking SEP Nordic walking SEP  Treadmill training: F - 3 days per week for 12 weeks  I - ACSM pain level 4 T - 50 minutes per session T - treadmill walking  Nordic walking:  F - 3 days per week for 12 weeks I - ACSM pain level 4  T - 50 minutes per session T - walking with Nordic poles under supervision | N/A |
| Cheetham | 2004 | UK | Circuit based SEP  SEP: F - 1 day per week for 12 months I - Self determined intensity by patient T - 45 minutes (5-10 min talk on benefits of exercise and 30 mins of exercise) T - Circuit walking with 2 min exercise stations; stair-climbing, low-step climbing, high-step climbing, tip-toe walking, standing on tip-toe from flat, standing on tip-toe from ankle dorsi-flexion and power-jogger walking.   They also enrolled on a 6-month motivational group | Basic exercise advice |
| Chehuen | 2017 | Brazil | Treadmill walking SEP Stretching based SEP  Treadmill SEP: F - 2 x week for 12 weeks I - HR at the pain threshold determined at baseline (±4bpm) T - 15 x 2 minutes bouts of walking separated by 2 minutes of upright rest, resulting in 30 minutes of effective intervention time. T - treadmill walking with the speed set at 2.0mp/h and gradient adjusted to keep HR at the target value.  Stretching based SEP: F – 2 days per week for 12 weeks T - 20 stretching exercises for all the main body muscles, executed 2 or three times maintaining the maximal stretch for 20 seconds. stated as 30mins. T - Stretching | N/A |
| Christman | 2003 | USA | HEP  An individualized exercise prescription was given to every intervention group member with initial frequency and duration based on physical ability, exercise history, and access to an exercise location. However, each prescription was based on three primary components:  F – 3 days per week for 12 weeks I - To maximal claudication pain T - At least 30 mins a session T - Walking  Also an education component; a 12-week, one-hour per week group education session. The average class size was four people, but one class had only two participants. Participants received a folder of material developed by the nurse investigator teaching the course, and contained information related to weekly course content. Each week, principles of exercise and smoking cessation were reviewed in addition to scheduled, weekly topics including (but not limited to): the natural history of claudication, controlling cholesterol, understanding nutrition labels, foot care for PAD patients, understanding and controlling hypertension, and understanding and controlling diabetes. | Usual care |
| Collins | 2011 | USA | HEP  F - 1 day per week with study instructor and at least 3 days per week by themselves I - Increase steps by 50 each session, no intensity reported T - 50 mins for each session T - Walking | Usual care control group |
| Collins | 2003 | USA | Pole striding SEP ± Vitamin E  F – 3 x week for 24 weeks I - Ranged from 70-80% measured maximal HR based on information obtained from the wheelchair and treadmill tests T - 45-60 minutes interval training not including rest - based on info obtained from the wheelchair and treadmill tests T - pole striding | Vitamin E alone or placebo alone  Control group came to the laboratory biweekly for ankle-brachial index (ABI) measurements. For the first 3 months, non-exercising subjects were seen biweekly by the study staff and monthly thereafter. During each visit body weight, heart rate, BP, and ABI were measured, a PAR completed, and drug compliance assessed. Data obtained from these visits were used for clinical purposes only. |
| Crowther | 2008 | Australia | Treadmill walking SEP  F - 3 days per week for 12 months I - Pain score of 3 or 4 T - Initially 25 minutes up to 40 minutes T - Treadmill walking | Usual care control group *Note they also included a healthy control group |
| Crowther(b) | 2012 | Australia | Treadmill walking SEP  F - 3 days per week for 6 months I - Pain score of 3 or 4 T - Initially 25 minutes up to 40 minutes T - Treadmill walking | Usual care control group |
| Cucato | 2013 | Brazil | Treadmill walking SEP Stretching based SEP  Walking Training:  F – 2 days per week for 12 weeks I - HR of claudication onset pain (within 4 beats) T - 15 x 2 minute bouts with 2 min rest T - Treadmill walking  Control "stretching exercise" F- 2 days per week for 12 weeks I - Not known T - 30 minutes per session T - Stretching exercises of upper and lower limbs | N/A |
| Delaney | 2014 | Australia | Treadmill walking SEP Treadmill walking and resistance SEP  Treadmill training: F - 2 days per week for 12 weeks I - walk till pain is unbearable T - 60 minutes per session T - treadmill walking   Resistance/Walking F - 2 days per week for 12 weeks I - Minimum level of resistance, increased by 5% each time they could complete 12 reps/sets T - 60 minutes per session T - resistance training on machines and treadmill walking | N/A |
| Gardner | 2001 | USA | Treadmill walking SEP  F - 3x a week for 6 months I - 50-80% of the maximal workload to near-maximal claudication pain T - Walking duration began at 15 minutes and was increased 5/minutes a month up to 40 minutes T - intermittent treadmill walking | Usual care control group  The control group did not receive any recommendations regarding exercise |
| Gardner | 2002 | USA | 12-month maintenance SEP from Gardner 2001 study.  6 month programme: F – 3 days per week for 6 months I - to near maximal pain starting at 50% of the maximal workload achieved during a maximal effort treadmill test, increasing on an individual basis to 80% by the sixth month T - Duration began at 15 minutes and progressed 5 minutes per month until 40 minutes was achieved by the sixth month. T - Intermittent TM walking  12 month maintenance programme: F – 2 days per week for 12 months I - 80% of the maximal workload T - 40 minutes T - Intermittent treadmill walking | Usual Care control group  The control group did not receive any recommendations regarding exercise |
| Gardner | 2005 | USA | High or low intensity treadmill walking SEP  HI SEP:  F - 3 days per week for 6 months I - A constant intensity of 80% of the maximum WL from the baseline maximal effort TM test T - Individualised to match the caloric expenditure that would have been accomplished if they had been randomised to the LI group. This on average started at 12 minutes, increasing to 35 minutes during month 6 T - Intermittent treadmill walking  LI SEP: F - 3 days per week for 6 months I - A constant intensity of 40% of the maximum WL from the baseline maximal effort TM test T - Started at 15 minutes and progressively increased by 5 minutes every month until 40 minutes T - Intermittent treadmill walking | N/A |
| Gardner | 2011 | USA | Treadmill walking SEP  HEP  SEP:  F - 3 days per week for 12 weeks I - 40% of final workload achieved at baseline to near-maximal claudication pain  T - Walking duration began at 15 minutes for the first 2 weeks of the program and progressively increased by 5 minutes biweekly until a total of 40 minutes of walking was accomplished during the final 2 weeks of the program T - intermittent treadmill walking  HEP -  F - 3 days per week for 12weeks I - To near-maximal claudication pain at a self-selected pace T - Started at 20 minutes and increased by 5 mins biweekly until 45 minutes during the final 2 weeks. These durations were 5 minutes longer than SEP in an attempt to better match the programmes on total volume of exercise. T - Intermittent walking | Usual care control group  All patients were advised to give up smoking and our regular clinical recommendations for risk factor management were followed.   Patients randomised to invasive therapy were referred for standard angiography. Based on the angiographic findings, the appropriate intervention, either an endovascular or open surgical procedure was determined according to current surgical management policy. |
| Gardner | 2012 | USA | Treadmill walking SEP  SEP:  F – 3 days per week for 6 months I - Began at an initial grade of 50% of the final workload attained during the baseline graded TM test and increased by 10% every 6 weeks up to 80% during the final 6 weeks of the programme. During each exercise session, patients walked at approximately 2 mph until their claudication pain reached a level of 3 on a 0 to 4 pain scale after which they rested until claudication pain completely resolved T - began at 15 minutes for the first month, increasing by 5 minutes per month until 40 minutes was accomplished T - Intermittent Treadmill walking | Usual care control group  Patients randomized to this group were encouraged to walk more on their own but did not receive specific recommendations regarding an exercise program during the study. |
| Gardner | 2014 | USA | Treadmill walking SEP  Resistance training SEP  HEP  SEP: F - 3 days per week for 12 weeks I - 40% of final workload achieved at baseline to near-maximal claudication pain  T - Walking duration began at 15 minutes for the first 2 weeks of the program and progressively increased by 5 minutes biweekly until a total of 40 minutes of walking was accomplished during the final 2 weeks of the program T - intermittent treadmill walking  HEP:  F - 3 days per week for 12 weeks I - To near-maximal claudication pain at a self-selected pace T - Started at 20 minutes and increased by 5 mins biweekly until 45 mins during the final 2 weeks. These durations were 5 mins longer than SEP in an attempt to better match the programmes on total volume of exercise. T - Intermittent walking  Resistance Programme: F – 3 days per week for 12 weeks I - resistance that caused fatigue within 15-reps (15-rep max), which was reassessed each month T - 1 set of 15 reps for 9 exercises T - light resistance training (The resistance training phase consisted of performing upper extremity exercises that included the bench press, military press, butterfly, biceps curl, triceps press-down, and lat pull- down. Lower extremity exercises included the leg press, leg curl, and leg extension). If the resistance from the exercise machine could not be lifted, resistance bands were used instead. | N/A |
| Hobbs | 2006 | UK | Circuit based SEP  F – 2 days per week for 12 weeks I - Moderate intensity in accordance with the ACSM guidelines T - 60 minutes T - A circuit of exercises - This sequence involved a shuttle walk at moderate to hard pace; paired toe raising to heel raising, with chair support for balance; continuous sitting and standing from chair; spot marching with high knees, swinging arms; arm exercises from the upright sitting position (side arm raising, alternate arm vertical pushing, and side bent-arm lift); shuttle walk at moderate to hard pace; knee bends with chair support for balance; alternate heel raising (left/right) with chair sup- port for balance; step-ups on bench or stairs; and arm exercises from the upright sitting position (straight side arm small backward circling, double arm vertical pushing, and side bent-arm lift, alternating arms). Subjects underwent 3 minutes of activity at each station followed by 2 minutes of rest.  On days when not attending the supervised sessions, subjects were asked to perform the exercise program unsupervised at home. Training logs were kept to detail the number of repetitions performed and the maximal heart rate at each station to ensure an adequate training effect. | BMT ± revascularisation  BMT = Smoking cessation, statin and antiplatelet therapy, screening for DM, BP control, Exercise and potentially Cilostazol.  BMT + PTA = unilateral infrainguinal PTA |
| Hobbs | 2007 | UK | Circuit based SEP  F – 2 days per week for 12 weeks I - Moderate intensity in accordance with the ACSM guidelines T - 60 minutes T - A circuit of exercises - This sequence involved a shuttle walk at moderate to hard pace; paired toe raising to heel raising, with chair support for balance; continuous sitting and standing from chair; spot marching with high knees, swinging arms; arm exercises from the upright sitting position (side arm raising, alternate arm vertical pushing, and side bent-arm lift); shuttle walk at moderate to hard pace; knee bends with chair support for balance; alternate heel raising (left/right) with chair sup- port for balance; step-ups on bench or stairs; and arm exercises from the upright sitting position (straight side arm small backward circling, double arm vertical pushing, and side bent-arm lift, alternating arms). Subjects underwent 3 minutes of activity at each station followed by 2 minutes of rest.  In addition to the supervised sessions, subjects were provided with a videotape of the exercise program and encouraged to undertake the exercises at home and complete an exercise log on the days that they did not attend the classes | BMT ± Cilostazol  BMT = Smoking cessation, statin and antiplatelet therapy, screening for DM, BP control, Exercise and potentially Cilostazol.  BMT + Cilostazol |
| Hodges | 2008 | UK | Treadmill walking SEP  F – 2 days per week for 12 weeks I - 75% of the initial grade achieved during the exercise test until they reached stage 3 or 4 of the PAD pain scale T - Until patient had accrued 30 minutes of exercise and each session lasted approx. 45 mins. T - Intermittent treadmill walking | Usual care control group  Patients were told to walk as often as possible but given no exercise regime to follow. |
| Jones | 1996 | USA | Treadmill walking SEP  Stairmaster SEP  F – 2 days per week for 12 weeks I - Patients exercised at the highest level attained and sustained for at least 1 minute on the progressive exercise test on their training modality severe pain (3/4) T - 60 minutes with 5-7 exercise/rest cycles T - Treadmill walking or stair master training | N/A |
| Kakkos | 2005 | UK | Treadmill walking SEP HEP  SEP: F - 3 x week for 6m I - to moderately severe pain T - 60 minutes T - Intermittent treadmill walking  HEP:  F - Daily I - to near maximal pain T - at least 45 minutes T - walking | Intermittent foot and calf pneumatic compression |
| Kropielnicka | 2018 | Poland | Treadmill walking SEP Nordic walking SEP Nordic walking + resistance training SEP  Treadmill walking: F - 3 days per week for 12 weeks I - submaximal pain 4/5 T - 45 minutes T - treadmill walking  Nordic walking: F - 3 days per week for 12 weeks I - submaximal pain 4/5 T - 45 minutes T - Nordic over ground walking  Nordic walking + resistance training: F - 3 days per week for 12 weeks, alternating between each modality per session I - submaximal pain 4/5 or 70-80 Fmax  T - 45 minutes, 100/200 reps per legs  T - over ground walking, isokinetic dynamometer plantar and dorsiflexion and sometimes knee flexion and extension | N/A |
| Lamberti | 2015 | Italy | HEP  F - 6 days per week 4 months  I - individually prescribed step rate T - 10 min x 2 T - treadmill or regular walking | Endovascular procedure |
| Langbein | 2002 | USA | Pole striding SEP  F - The pole striding exercise program consisted of supervised training 3 times per week for 4 weeks, twice per week for 8 weeks, once per week for 4 weeks, biweekly for 4 weeks, and unsupervised training (4-5 x per week) for 4 weeks. I - submaximal pain 4/5 T – not reported T - pole striding | Usual care control group  Continued to receive standard medical care. To control for attention bias, subjects were seen biweekly by the study staff for the first 3 months and monthly thereafter.  During each visit, body weight, HR, BP, and ABI were measured. |
| Leicht | 2011 | Australia | Treadmill walking SEP  F - 3 days per week for 12 months I - maximal pain T - 25-40 min T - treadmill walking | Conservative management |
| Maejima | 2005 | Japan | Overground walking SEP ± heparin  SEP or SEP + heparin F - daily I - until patient noted claudication T - 60 minutes T - over ground walking | Daily heparin injections |
| Mays | 2015 | USA | Treadmill based SEP  Community-based Walking group: F = 3x pw for 14 weeks I = moderate pain or moderate - hard on RPE (if no IC experienced) T = 25-50 minutes  T = treadmill and walking outside | Usual care control group |
| Mazari, | 2010 | UK | Circuit based SEP ± revascularisation  SEP  F = 3 days per week for 12 weeks  I = Not detailed  T = 24 = minutes  T = circuit training and walking in-between stations   PTA + SEP  F = 3 days per week for 12 weeks  I = Not detailed  T = 24 = minutes  T = circuit training and walking in-between stations | Revascularisation  PTA |
| McGuigan, | 2001 | USA | Resistance training SEP  F = 3 days per week for 24 weeks I = % 10RM T = not stated T = resistance exercises (dumbbells etc) | Usual care control group |
| Mika | 2006 | Poland | Treadmill walking SEP  F – 3 days per week for 12 weeks I - 85% ICD (no pain) T - 60 minutes T - treadmill walking | Usual care control group |
| Mika | 2011 | Poland | Treadmill walking SEP  F - 3 days per week for 12 weeks I - 2/5 pain of the pain scale T - 30-55 minutes T - treadmill walking | Usual care control group  All encouraged to stop smoking |
| Mika | 2013 | Poland | Pain free treadmill walking SEP  Moderate pain treadmill walking SEP  SEP pain free: F - 3 days per week for 12 weeks I - 2/5 pain of the pain scale T - 35-60 minutes T - treadmill walking  SEP moderate pain: F - 3 days per week for 12 weeks  I - 4/5 pain of the pain scale  T - 35-60 minutes T - treadmill walking | N/A |
| Murphy | 2011 | USA | Treadmill walking SEP  F – 3 days per week for 6 months I - 3-4/5 pain of the pain scale T - 1 hour T - treadmill walking | BMT  revascularisation |
| Nicolai | 2010 | Netherlands | Circuit based SEP  F – 3 days per week for 12 months I - unclear  T - 20-30 minutes T - physical therapy including endurance and strength exercise" | Walking advice |
| Novakovic | 2019 | Slovenia | Treadmill walking pain free SEP  Treadmill walking moderate pain SEP  Pain free SEP:  F – 2-3 days per week for 12 weeks I – until claudication onset distance T – 60 minutes T – treadmill walking  Moderate pain SEP:  F – 2-3 days per week for 12 weeks I – until moderate pain (3-4/5) T – 60 minutes T – treadmill walking | Usual care control group  Advised to continue with secondary preventive activities, including regular walking, |
| Parmenter | 2013 | Australia | High intensity resistance training  Low intensity resistance training  F – 3 days per week for 6 months  H-RT was set at 50% of the participant’s peak strength determined at baseline (1repetition maximum (1RM)) and increased over four sessions until 80% 1RM was reached. Repetition maximum testing was repeated at 2-week intervals, and intensity was adjusted accordingly and fine-tuned according to a rating of between 15 and 18 on the Borg Rating of Perceived Exertion Scale14 for each set performed.  L-RT group the intensity (load and progression) used. The initial intensity for this group was set at 20% of their peak strength determined at baseline (20% 1RM) and increased each session in 2% increments until 30% baseline | Walking advice  Instructed to walk outside unsupervised at their usual walking speed to maximum pain levels tolerable, rest, and repeat for up to 30 minutes (not including rest) three times per week. |
| Parr | 2009 | RSA | Upper body strength training SEP  SEP  SEP: F – 3 days per week for 6 weeks I - onset of pain T - 45 minutes T - treadmill walking, cycling, strength, flexibility  Upper body strength:  F – 3 days per week for 6 weeks I - 15 repetitions of exercises on 10 upper body weight plated machines and 30  repetitions of 4 upper body dumbbell exercises (weight of 1.5kg). The initial weight was set so the patient could comfortably  complete 15 repetitions. Weights were increased between 4 and 16 pounds (1.8 and 7.3 kg) per week. | Walking advice |
| Patterson | 1997 | USA | Treadmill walking / cycling training SEP  HEP  SEP: F = 3 days per week for 12 weeks I = 75% of the previously determined MWT (taken from other source that was referenced in the methods) T = 60 minutes T = treadmill and leg ergometry and Air-Dyne cycling  HEP@ F = min 3 days per week for 12 weeks  I = walk to tolerance T = 20-40 minutes  T = walking | N/A |
| Regensteiner, | 1997 | USA | Treadmill walking SEP  HEP  SEP: F = 3 days per week for 12 weeks I = mild or moderate level of pain (scored as 3 or 4 on a 1-to-5 scale) T = 35 minutes plus T = treadmill walking  HEP: F = 3 days per week for 12 weeks  I = walking speed as rapid as can tolerate T = 35 minutes plus T = outdoor walking | N/A |
| Ritti-Dias | 2010 | Brazil | Resistance training SEP  Treadmill walking SEP  Strength Training: F - 2 days per week for 12 weeks I - RPE 11-13 on 15 point BORG T - 60 minutes T - Machines   Walk Training: F - 2 days per week for 12 weeks I - RPE 11-13 on 15 point BORG  T - 60 minutes T - Walking on a treadmill | N/A |
| Sandercock | 2007 | UK | Treadmill training SEP  HEP  SEP: F = 2 days per week for 12 weeks (+ one home-based walking) I = 70-75% VO2 peak T = 30 minutes in total T = treadmill walking  HEP: F = 3 days per week for 12 weeks I = RPE 12-14 T = 30 minutes in total T = walking outside | Usual care control group |
| Sanderson | 2006 | Australia | Cycle training SEP  Treadmill training SEP  Cycle: F = 3 days per week for 6 weeks I = 80% VO2 peak T = individualised T = cycle ergometer  Treadmill: F = 3 days per week for 6 weeks I = 80% VO2 peak T = individualised T = treadmill | Usual care control group |
| Savage | 2001 | USA | Treadmill walking SEP  HEP  SEP:  F = 3 days per week for 12 weeks  I = 60% max gradient, walk to intense pain  T = 15 minutes to 40 minutes  T = treadmill walking  HEP:  F = 3 days per week  I = intense pain  T = 15 minutes to 40 minutes  T = walking | N/A |
| Schlager | 2011 | Austria | Treadmill walking SEP  F - 2 days per week for 6 months  I - moderate claudication  T - 35 minutes progressed to 50 minutes  T - Not stated | Usual care control group |
| Spafford | 2014 | UK | Nordic pole walking HEP  HEP  Nordic Pole Walking:  F – at least 3 days per week for 12 weeks  I - not stated  T - 30 minutes  T - Nordic pole walking  HEP:  F = 3 days per week for 12 weeks  I - normal walking pace  T = 30 minutes  T = walking | N/A |
| Spronk | 2009 | Netherlands | Treadmill walking SEP then HEP.  SEP:  F - 2 x week for 24 weeks  I - To maximum claudication pain, treadmill walking at 3.5 km/h without a gradient  T - 30 minutes per session  T - Treadmill walking.  In addition, all patients were instructed to walk for at least 30 minutes three times a week outside the hospital setting.  HEP:  Patients were instructed to continue their walking exercise daily in their own environment without supervision and with claudication pain as a guide. Patients received tips for keeping their exercise motivation. | Endovascular revascularisation + given general recommendations concerning lifestyle changes according to the guidelines for cardiovascular disease prevention |
| Stewart | 2008 | UK | Circuit based SEP then HEP.  SEP:  F - 2 x week for 12 weeks  I - To maximum claudication pain  T - 1hr session  T - Circuit format 5 exercises mainly based on calf muscle.  HEP:  Patients continued with unsupervised exercise for a further 3 months. | Control group: exercise advice only for three months  HEP: Patients continued with unsupervised exercise for a further 3 months |
| Szymczak | 2016 | Poland | Treadmill training SEP and Resistance training SEP  Walking arm:  F - 2 x week for 12 weeks  I - Light claudication pain (3/5)  T - 50 minutes  T - Treadmill  Resistance Arm:  F - 2 x week for 12 weeks  I - 15 repetitions  T - 50 minutes  T - Seated resistance machines using various muscle groups | N/A |
| Tebbutt | 2011 | UK | Resisted plantar flexion exercise (plus standard care):  F - 3 x week for 12 weeks  I - 6kg resistance  T - 2 minutes exercise/2 minutes rest, 10 times, to equal 20 minutes of exercise in total  T - Step it rocking pedal Standard care:  Standard care - Patients advised to walk to their maximum walking distance each day and to attempt to increase this distance as they were able. | Control group (standard care)  Standard care: Patients advised to walk to their maximum walking distance each day and to attempt to increase this distance as they were able. |
| Tew | 2009 | UK | Arm crank SEP  F - 2 x week for 12 weeks  I - 60-70% of peak work rate achieved in the initial arm-crank assessment  T - 2-min exercise at a crank rate of 50 rev./min, followed by 2 min of rest, for a total exercise time of 20 min in a 40-min session  T - Arm-cranking | Usual care control group |
| Tew | 2015 | UK | HEP  Patients were encouraged to gradually build up their total daily steps to more than 7500,12 and to perform at least 2500 of these steps as ‘exercise steps’, which should approximate 30 minutes of walking assuming a mean step cadence of 90 steps/minute. For their ‘exercise steps’, participants are advised to walk at a speed that evokes a strong claudication pain and to persevere walking as long as is tolerable. | Usual care control group |
| Tisi | 1997 | UK | Circuit based SEP  F - Weekly classes for 4 weeks  I - Performed to the limit of claudication pain  T - 1hr  T - A series of active and passive leg exercises Patients were encouraged to exercise for at least 45 min every day at home, in addition to daily walks of at least 1 mile. | Revascularisation  Observation |
| Treat-Jacobson | 2009 | USA | Arm crank SEP  Treadmill walking SEP  Arm crank and treadmill walking SEP  All 3 interventions F - 3 x week for a total of 36 sessions.  Arm ergometry:  I - exercising at one work level (10 watts) below the maximal level achieved during their baseline arm-ergometry test at a rate of 50 cycles per minute  T - worked against this load intermittently for periods of 2 minutes of exercise, followed by 2 minutes of rest, for a total of up to 60 minutes  T - Arm ergometry  Treadmill walking:  I - initially began exercising at 2 mph (3.2 km/h) at a 0% grade. They walked until their claudication pain became moderately severe (4 of 5 on the claudication scale), and they then stopped, sat down, and rested until the pain subsided.  T - This exercise/rest cycle was repeated throughout each 60-minute exercise session.  T - Treadmill walking  Arm-ergometry and treadmill walking combination:  I - same as above  T - 20 minutes of arm ergometry and 40 minutes of treadmill adopting the same protocols as the previous groups.  T - Arm-ergometry and treadmill walking | Exercise advice |
| Tsai | 2002 | Taiwan | Treadmill walking SEP  F - 3 x week for 12 weeks  I - Started from 2 mph, 0% grade, with 1% grade increase every 10 min if patients reported a claudication pain score below 2. claudication pain  scores between 2 and 3 (pain levels between mild and moderate). | Usual care control group |
| Van Schaardenburgh | 2017 | Norway | Calf raise HEP  HEP  Calf raise group:  F - 3 x per day  I - Calf raises until pain was felt, then asked to perform 5 more repetitions  T - N/A  T - Body weight  Treadmill group:  F - 3 x week  I - instructed to walk near the pain threshold  T - 30 minutes  T - walking | N/A |
| Villemur | 2020 | France | Conventional treadmill walking SEP  Interval treadmill walking with active recovery SEP  Conventional training:  F - 5 x week for 4 weeks  I - Speed set a 3.2km/h with 0% slope for those unable to walk at this speed this was reduced to 1.6km/h, 0% slope  T - 50min  T - Constant load treadmill walking    ITAR:  F - 5 x week for 4 weeks.  I - Walking speed set at 70% and active recovery set at 40% of baseline.  T - 40min  T - Interval treadmill walking.  Both groups received an additional daily 2-hr supervised exercise | N/A |
| Walker | 2000 | UK | Arm crank SEP  Cycle training SEP  Lower limb:  F - 2 x week for 6 weeks  I - Penultimate workload achieved in the respective  assessment was used as the initial training intensity T - 2 minutes of exercise, followed by 2 minutes of rest, for a total exercise time of 20 minutes in a 40-minute session  T - lower limb cycle ergometer  Upper limb:  F - 2 x week for 6 weeks  I - Penultimate workload achieved in the respective assessment was used as the initial training intensity T - 2 minutes of exercise, followed by 2 minutes of rest, for a total exercise time of 20 minutes in a 40-minute session  T - upper limb cycle ergometer | Exercise advice |
| Wang | 2008 | Norway | Plantar flexion SEP  Individual leg plantar flexion interval training:  F - 3 x week for 8 weeks  I - Initial workload was set at 80% of maximum work rate. As work capacity increased the resistance toward movement was increased, by increments of 1 W, to maintain the work intensity. T - 4-min work periods for the individual leg, and 4 intervals were executed on each leg. One leg rested while the other performed work. 40 mins total  T- Plantar flexion ergometer. | Usual care control group |
| Wood | 2006 | Australia | Treadmill walking SEP  F - 3 x week for 6 weeks I - Training intensity for the first 3 weeks was equal to the walking speed that elicited 80% peak V ̇ o2 achieved during the initial incremental walking test. For the final 3 weeks, the intensity was increased to a walking speed corresponding to 100% peak V ̇ o2. T - 40 mins T - Treadmill Patients were asked to continue their normal daily activities. | Usual care control group |
| Zwierska | 2005 | UK | Arm crank SEP  Cycle training SEP  F - 2 x week for 24 weeks  I - penultimate power output enabled training intensity to be balanced at 85% to 90% of the limb-specific peak VO2  T - 2 minutes exercise at a crank rate of 50 rev/min, followed by 2 minutes rest, for a total exercise time of 20 minutes in a 40-minute session.  T- electronically braked cycle ergometer | Usual care control group |
| BMT = best medical therapy, HR = Heart rate, HEP = Home exercise programme, SEP = Supervised exercise programme, F = Frequency, I = Intensity, T = Time, T = Type. | | | | |
