# Supplementary material for: A systematic review of exercise testing in patients with intermittent claudication: A focus on test standardisation and reporting quality in randomised controlled trials of exercise interventions
Source: PLoS One. 2021 May 3;16(5):e0249277. doi: 10.1371/journal.pone.0249277 (PMC8092776; doi:10.1371/journal.pone.0249277)
Supplement: S2 Table — (DOCX) [file pone.0249277.s003.docx]

S2 Table: Medline search terms.

1. Exp. Peripheral vascular diseases
2. Exp. Peripheral arterial disease
3. Exp. Intermittent claudication
4. Maximum walking distance
5. Maximal walking distance
6. MWD
7. Maximal walking time
8. Maximum walking time
9. Exp. Exercise
10. Exp. Exercise therapy
11. Exp. Rehabilitation
12. Walking therapy
13. Treadmill
14. Training
15. Fitness
16. Plantar Flexion
17. Absolute Claudication Distance
18. ACD
19. MWT
20. Maximum claudication distance
21. Maximal claudication distance
22. Maximal claudication time
23. Maximum claudication time
24. MCT
25. MCD
26. Absolute claudication time
27. ACT
28. 1 OR 2 OR 3
29. 4 OR 5 OR 6 OR 7 OR 8 OR 17 OR 18 OR 19 OR 20 OR 21 OR 22 OR 23 OR 24 OR 25 OR 26 OR 27
30. 9 OR 10 OR 11 OR 12 OR 13 OR 14 OR 15 OR 16
31. 28 AND 29 AND 30
