# Supplementary material for: A systematic review of exercise testing in patients with intermittent claudication: A focus on test standardisation and reporting quality in randomised controlled trials of exercise interventions
Source: PLoS One. 2021 May 3;16(5):e0249277. doi: 10.1371/journal.pone.0249277 (PMC8092776; doi:10.1371/journal.pone.0249277)
Supplement: S3 Table — (DOCX) [file pone.0249277.s004.docx]

S3 Table. Implementation and reporting quality for treadmill testing

|  | **Test Equipment & Protocol** | | **Pre-test** | | | | **Conducting the test** | | | | | **T** |
| --- | --- | --- | --- | --- | --- | --- | --- | --- | --- | --- | --- | --- |
|  | Equipment calibrated | Cites & correctly implements protocol | Clearly states PT’s were rested | Clearly states PT’s was fasted, avoided cig and alcohol | IC pain scale was used | MWC as sole termination criteria | Clearly states qualification level of administrator | Clearly states familiarisation to protocol | Clearly states treadmill screen/timer was hidden | Clearly states If handrail support was permitted | Clearly states PT’s achieved maximal claudication |  |
| Allen, 2010 | 0 | 0 | 0 | 0 | 0 | 1 | 0 | 0 | 0 | 0 | 0 | **1** |
| Baker, 2017 | 0 | 1 | 0 | 0 | 0 | 1 | 0 | 1 | 0 | 0 | 0 | **3** |
| Brenner, 2017 | 0 | 0 | 0 | 0 | 0 | 0 | 0 | 0 | 0 | 0 | 0 | **0** |
| Bronas, 2011 | 0 | 0 | 1 | 1 | 0 | 1 | 0 | 1 | 0 | 1 | 1 | **6** |
| Bulinska, 2015 | 0 | 1 | 0 | 0 | 1 | 0 | 0 | 1 | 0 | 0 | 0 | **3** |
| Cheetham, 2004 | 0 | 0 | 0 | 0 | 0 | 1 | 0 | 1 | 0 | 0 | 0 | **2** |
| Collins, 2011 | 0 | 1 | 0 | 0 | 0 | 1 | 0 | 0 | 0 | 0 | 0 | **2** |
| Crowther, 2008 | 0 | 1 | 0 | 0 | 1 | 0 | 0 | 0 | 0 | 1 | 0 | **3** |
| Crowther, 2012 | 0 | 1 | 1 | 0 | 1 | 0 | 0 | 0 | 0 | 0 | 0 | **3** |
| Ritti-Dias 2010 | 0 | 1 | 1 | 0 | 0 | 1 | 1 | 1 | 0 | 0 | 0 | **5** |
| Cucato, 2013 | 0 | 1 | 0 | 0 | 0 | 1 | 0 | 0 | 0 | 0 | 0 | **2** |
| Schlager, 2011 | 0 | 1 | 0 | 0 | 0 | 0 | 1 | 0 | 0 | 0 | 0 | **2** |
| Savage, 2001 | 0 | 1 | 0 | 0 | 0 | 1 | 0 | 0 | 0 | 0 | 0 | **2** |

Table 3 cont

|  | **Test Equipment & Protocol** | | **Pre-test** | | | | **Conducting the test** | | | | | **T** |
| --- | --- | --- | --- | --- | --- | --- | --- | --- | --- | --- | --- | --- |
|  | Equip-ment calibrated | Cites & correctly implements protocol | Clearly states PT’s were rested | Clearly states PT’s was fasted, avoided cig and alcohol | IC pain scale was used | MWC as sole termination criteria | Clearly states qualification level of administrator | Clearly states familiarisation to protocol | Clearly states treadmill screen/timer was hidden | Clearly states If handrail support was permitted | Clearly states PT’s achieved maximal claudication |  |
| Sanderson, 2006 | 0 | 0 | 0 | 0 | 1 | 1 | 0 | 1 | 0 | 0 | 1 | **4** |
| Sandercock, 2007 | 0 | 1 | 0 | 0 | 1 | 0 | 0 | 1 | 0 | 0 | 0 | **3** |
| Regenstein-er, 1997 | 0 | 1 | 1 | 0 | 1 | 1 | 1 | 1 | 0 | 0 | 0 | **6** |
| Patterson, 1997 | 0 | 0 | 0 | 0 | 0 | 1 | 0 | 1 | 0 | 0 | 1 | **3** |
| McGuigan, 2001 | 0 | 0 | 0 | 0 | 0 | 1 | 0 | 0 | 0 | 0 | 0 | **1** |
| Mazari, 2010 | 0 | 0 | 0 | 0 | 0 | 0 | 0 | 0 | 0 | 0 | 0 | **0** |
| Mays, 2015 | 0 | 1 | 0 | 0 | 1 | 0 | 0 | 0 | 0 | 0 | 0 | **2** |
| Kropielnicka, 2018 | 0 | 0 | 0 | 0 | 0 | 0 | 1 | 1 | 0 | 0 | 0 | **2** |
| Lamberti, 2015 | 0 | 0 | 0 | 0 | 0 | 0 | 1 | 0 | 0 | 0 | 0 | **1** |
| Langbein, 2002 | 0 | 0 | 0 | 0 | 0 | 1 | 0 | 1 | 0 | 0 | 0 | **2 (G)** |
|  | 0 | 0 | 0 | 0 | 0 | 1 | 0 | 0 | 0 | 0 | 0 | **1 (CW)** |
| Leicht, 2011 | 0 | 1 | 1 | 0 | 0 | 0 | 0 | 0 | 0 | 0 | 0 | **2** |
| Maejima, 2005 | 0 | 0 | 0 | 0 | 0 | 1 | 0 | 0 | 0 | 0 | 0 | **1** |
| Mika, 2006 | 1 | 1 | 0 | 0 | 1 | 1 | 1 | 1 | 0 | 0 | 0 | **6** |

Table 3 cont

|  | **Test Equipment & Protocol** | | **Pre-test** | | | | **Conducting the test** | | | | | **T** |
| --- | --- | --- | --- | --- | --- | --- | --- | --- | --- | --- | --- | --- |
|  | Equip-ment calibrated | Cites & correctly implements protocol | Clearly states PT’s were rested | Clearly states PT’s was fasted, avoided cig and alcohol | IC pain scale was used | MWC as sole termination criteria | Clearly states qualification level of administrator | Clearly states familiarisation to protocol | Clearly states treadmill screen/timer was hidden | Clearly states If handrail support was permitted | Clearly states PT’s achieved maximal claudication |  |
| Mika, 2011 | 1 | 1 | 0 | 0 | 1 | 1 | 1 | 1 | 0 | 1 | 0 | **7** |
| Mika, 2013 | 1 | 1 | 0 | 0 | 1 | 1 | 1 | 1 | 0 | 1 | 0 | **7** |
| Murphy, 2012 | 0 | 1 | 0 | 0 | 0 | 1 | 0 | 1 | 0 | 0 | 0 | **3** |
| Nicolai, 2010 | 0 | 1 | 0 | 0 | 0 | 1 | 0 | 0 | 0 | 0 | 0 | **2** |
| Novakovic, 2019 | 0 | 0 | 0 | 0 | 1 | 1 | 0 | 0 | 0 | 0 | 0 | **2** |
| Parr, 2009 | 0 | 0 | 0 | 0 | 1 | 1 | 0 | 0 | 0 | 0 | 0 | **2** |
| Chehuen, 2017 | 0 | 1 | 0 | 1 | 0 | 1 | 1 | 1 | 0 | 0 | 0 | **5** |
| Christman, 2003 | 0 | 0 | 0 | 0 | 0 | 1 | 1 | 0 | 0 | 0 | 0 | **2** |
| Collins, 2003 | 0 | 0 | 0 | 0 | 0 | 1 | 1 | 1 | 0 | 0 | 1 | **4 (G)** |
|  | 0 | 0 | 0 | 0 | 0 | 1 | 1 | 0 | 0 | 0 | 1 | **3 (CW)** |
| Gardner, 2001 | 0 | 1 | 0 | 0 | 0 | 1 | 0 | 1 | 0 | 1 | 0 | **4** |
| Gardner, 2002 | 0 | 1 | 0 | 0 | 0 | 1 | 0 | 1 | 0 | 1 | 0 | **4** |
| Gardner, 2005 | 0 | 1 | 1 | 0 | 0 | 1 | 0 | 0 | 0 | 0 | 0 | **3** |

Table 3 cont

|  | **Test Equipment & Protocol** | | **Pre-test** | | | | **Conducting the test** | | | | | **T** |
| --- | --- | --- | --- | --- | --- | --- | --- | --- | --- | --- | --- | --- |
|  | Equip-ment calibrated | Cites & correctly implements protocol | Clearly states PT’s were rested | Clearly states PT’s was fasted, avoided cig and alcohol | IC pain scale was used | MWC as sole termination criteria | Clearly states qualification level of administrator | Clearly states familiarisation to protocol | Clearly states treadmill screen/timer was hidden | Clearly states If handrail support was permitted | Clearly states PT’s achieved maximal claudication |  |
| Gardner, 2011 | 0 | 0 | 0 | 0 | 0 | 1 | 0 | 1 | 0 | 0 | 0 | **2** |
| Gardner, 2011 | 0 | 1 | 0 | 0 | 0 | 1 | 0 | 0 | 0 | 0 | 0 | **2** |
| Gardner, 2014 | 0 | 1 | 0 | 0 | 0 | 0 | 0 | 1 | 0 | 0 | 0 | **2** |
| Hobbs, 2006 | 0 | 0 | 1 | 1 | 0 | 1 | 0 | 1 | 0 | 0 | 0 | **4** |
| Hobbs, 2007 | 0 | 0 | 1 | 1 | 0 | 1 | 0 | 0 | 0 | 0 | 0 | **3** |
| Hodges, 2008 | 0 | 0 | 1 | 1 | 1 | 0 | 0 | 0 | 0 | 1 | 1 | **5** |
| Kakkos, 2005 | 0 | 0 | 0 | 0 | 0 | 1 | 0 | 1 | 0 | 0 | 0 | **2** |
| Spronk, 2009 | 0 | 0 | 0 | 0 | 0 | 1 | 0 | 0 | 0 | 0 | 0 | **1** |
| Stewart, 2008 | 0 | 0 | 0 | 0 | 0 | 0 | 0 | 1 | 1 | 0 | 1 | **3** |
| Szymczak,2016 | 0 | 0 | 0 | 0 | 0 | 0 | 0 | 0 | 0 | 0 | 0 | **0** |
| Tebbutt, 2011 | 0 | 0 | 0 | 0 | 0 | 0 | 0 | 0 | 0 | 0 | 1 | **1** |
| Tew, 2009 | 0 | 0 | 0 | 1 | 0 | 0 | 0 | 1 | 0 | 0 | 0 | **2** |
| Tew, 2015 | 0 | 1 | 0 | 0 | 0 | 1 | 0 | 0 | 0 | 0 | 0 | **2** |

Table 3 cont

|  | **Test Equipment & Protocol** | | **Pre-test** | | | | **Conducting the test** | | | | | **T** |
| --- | --- | --- | --- | --- | --- | --- | --- | --- | --- | --- | --- | --- |
|  | Equip-ment calibrated | Cites & correctly implements protocol | Clearly states PT’s were rested | Clearly states PT’s was fasted, avoided cig and alcohol | IC pain scale was used | MWC as sole termination criteria | Clearly states qualification level of administrator | Clearly states familiarisation to protocol | Clearly states treadmill screen/timer was hidden | Clearly states If handrail support was permitted | Clearly states PT’s achieved maximal claudication |  |
| Treat-Jacobson, 2009 | 0 | 0 | 0 | 1 | 1 | 1 | 1 | 1 | 0 | 1 | 0 | **6** |
| Tsai, 2002 | 0 | 0 | 0 | 0 | 1 | 1 | 0 | 0 | 0 | 0 | 1 | **3** |
| Van Schaardenburgh, 2017 | 0 | 0 | 0 | 0 | 0 | 0 | 0 | 0 | 0 | 0 | 0 | **0** |
| Villemur, 2020 | 0 | 0 | 1 | 0 | 0 | 1 | 1 | 0 | 0 | 1 | 0 | **4** |
| Wang, 2008 | 0 | 1 | 0 | 0 | 0 | 1 | 0 | 0 | 0 | 0 | 0 | **2** |
| Wood, 2006 | 0 | 1 | 0 | 0 | 0 | 1 | 0 | 0 | 0 | 0 | 0 | **2** |
| Jones, 1996 | 0 | 0 | 1 | 0 | 0 | 1 | 0 | 0 | 0 | 0 | 0 | **2 (G)** |
|  | 0 | 0 | 1 | 0 | 0 | 1 | 0 | 0 | 0 | 0 | 0 | **2 (CW)** |
| Tisi, 1997 | 0 | 0 | 1 | 0 | 0 | 1 | 0 | 1 | 0 | 0 | 0 | **3** |

Full description of the treadmill testing criteria is in table 1. PT’s, patient’s; cig, cigarettes; IC, intermittent claudication; MWC, maximal walking capacity; T, total
