# Supplementary material for: A systematic review of exercise testing in patients with intermittent claudication: A focus on test standardisation and reporting quality in randomised controlled trials of exercise interventions
Source: PLoS One. 2021 May 3;16(5):e0249277. doi: 10.1371/journal.pone.0249277 (PMC8092776; doi:10.1371/journal.pone.0249277)
Supplement: S4 Table — (DOCX) [file pone.0249277.s005.docx]

S4 Table. Implementation and reporting quality for corridor testing

|  | **Test Equipment & Protocol** | | **Pre-test** | | | | | | | **Conducting the test** | | | |  |
| --- | --- | --- | --- | --- | --- | --- | --- | --- | --- | --- | --- | --- | --- | --- |
|  | Equip-ment calibrated | Cites protocol or accepted guidelines | Corridor length stated | Clearly states PT’s avoided vig. ex 24hrs prev | Clearly states PT’s avoided cig and alcohol | IC pain scale was used | Clearly states PT’s were rested prior test | Clearly states standardised instructions were given | MWC as sole termination criteria | Clearly states qualification level of administrator | Clearly states familiarisation to protocol | Clearly states standardised verbal phrases | Clearly states PT’s achieved maximal claudication | **T** |
| Bulinska, 2015 | N/A | 1 | 1 | 1 | 1 | 0 | 1 | 1 | N/A | 1 | 1 | 1 | N/A | **9** |
| Delaney, 2014 | N/A | 0 | 0 | 0 | 0 | 0 | 1 | 0 | N/A | 0 | 0 | 0 | N/A | **1** |
| Spafford, 2014 | 0 | 0 | 0 | 0 | 0 | 1 | 0 | 0 | 1 | 0 | 0 | 0 | 1 | **3** |
| McGuigan, 2001 | N/A | 1 | 1 | 0 | 0 | 0 | 0 | 1 | N/A | 0 | 0 | 0 | N/A | **3** |
| Kropielnic-ka, 2018 | N/A | 1 | 1 | 1 | 1 | 0 | 1 | 1 | N/A | 1 | 1 | 1 | N/A | **9** |
| Lamberti, 2015 | N/A | 1 | 0 | 0 | 0 | 0 | 0 | 1 | N/A | 1 | 0 | 0 | N/A | **3** |
| Parmenter, 2013 | N/A | 1 | 0 | 0 | 0 | 0 | 0 | 0 | N/A | 0 | 1 | 0 | N/A | **2** |
| Parr, 2009 | N/A | 0 | 0 | 0 | 0 | 1 | 1 | 1 | N/A | 0 | 0 | 0 | N/A | **3** |
| Gardner, 2001 | N/A | 1 | 0 | 0 | 0 | 0 | 0 | 0 | N/A | 1 | 0 | 0 | N/A | **2** |
| Gardner, 2002 | N/A | 1 | 0 | 0 | 0 | 0 | 0 | 0 | N/A | 1 | 0 | 0 | N/A | **2** |
| Gardner, 2005 | N/A | 1 | 0 | 0 | 0 | 0 | 0 | 0 | N/A | 1 | 0 | 0 | N/A | **2** |
| Gardner 2011 | N/A | 1 | 1 | 0 | 0 | 0 | 0 | 1 | N/A | 1 | 0 | 0 | N/A | **4** |
| Gardner, 2014 | N/A | 1 | 0 | 0 | 0 | 0 | 0 | 0 | N/A | 0 | 0 | 0 | N/A | **1** |
| Szyczak, 2016 | N/A | 0 | 0 | 0 | 0 | 0 | 0 | 1 | N/A | 0 | 0 | 0 | N/A | **1** |
| Tew, 2015 | N/A | 1 | 0 | 0 | 0 | 0 | 0 | 0 | N/A | 0 | 0 | 0 | N/A | **1** |

Table 4 cont

|  | **Test Equipment & Protocol** | | **Pre-test** | | | | | | | **Conducting the test** | | | |  |
| --- | --- | --- | --- | --- | --- | --- | --- | --- | --- | --- | --- | --- | --- | --- |
|  | Equip-ment calibrated | Cites protocol or accepted guidelines | Corridor length stated | Clearly states PT’s avoided vig. ex 24hrs prev | Clearly states PT’s avoided cig and alcohol | IC pain scale was used | Clearly states PT’s were rested prior test | Clearly states standardised instructions were given | MWC as sole termination criteria | Clearly states qualification level of administrator | Clearly states familiarisation to protocol | Clearly states standardised verbal phrases | Clearly states PT’s achieved maximal claudication | **T** |
| Tsai, 2002 | N/A | 0 | 0 | 0 | 0 | 0 | 0 | 1 | N/A | 1 | 1 | 0 | N/A | **3** |
| Van Schaardenburgh, 2017 | N/A | 1 | 1 | 1 | 1 | 0 | 1 | 1 | N/A | 1 | 1 | 1 | N/A | **9** |
| Villemur, 2020 | N/A | 1 | 1 | 1 | 1 | 0 | 1 | 1 | N/A | 1 | 1 | 1 | N/A | **9** |
| Walker 2000 | 0 | 1 | 0 | 0 | 0 | 0 | 0 | 0 | 1 | 0 | 0 | 0 | 0 | **2** |
| Zwierska, 2005 | 0 | 1 | 0 | 1 | 1 | 0 | 0 | 0 | 1 | 0 | 1 | 0 | 0 | **5** |

Full description of the treadmill testing criteria is in table 2. PT’s, patient’s; cig, cigarettes; vig, vigorous; ex, exercise; prev, previously; IC, intermittent claudication; MWC, maximal walking capacity; N/A, not applicable; T, total
