# Supplementary material for: A systematic review of exercise testing in patients with intermittent claudication: A focus on test standardisation and reporting quality in randomised controlled trials of exercise interventions
Source: PLoS One. 2021 May 3;16(5):e0249277. doi: 10.1371/journal.pone.0249277 (PMC8092776; doi:10.1371/journal.pone.0249277)
Supplement: Attachment — Submitted filename: Response to Reviewers.docx [file pone.0249277.s007.docx]

Mr Sean Pymer

Academic Vascular Surgical Unit

Hull York Medical School

Hull, UK

**Dear Dr. Abaraogu**

**RE: Manuscript ID: PONE-D-20-40047**

We wish to thank the reviewers, Chris Seenan and Jibril Mohammed, for their time and efforts in reviewing our manuscript. You kindly summarised the main points from the reviewers, with no additional comments, so we hope that by responding to the reviewer’s comments, we will also have covered the points you raised.

**Reviewer #1:**

**Comment:** Exclusion of studies other than on exercise interventions. Perhaps the authors could review the methods to clearly state the rationale for exclusion of non-exercise intervention RCTs or re-run the search/ include studies of interventions other than exercise. Exercise testing is used in PAD and IC research for multiple interventions, and this may allow a more comprehensive summary of the current use of exercise testing in PAD and IC research. If not including/ changing methods, then this should be addressed in the limitations section.

**Response:** We absolutely agree with this comment, however, we felt that if we included these studies, the review would have been unmanageable and potentially overwhelming for the reader. We also felt that the addition of these studies would not alter our findings. We have therefore added some additional detail in the methods section “exclusion of these studies, and non-RCT's, is in line with a previous review on reporting standards and also ensured that the current review and the number of included studies was manageable” and the limitations section as suggested, “finally, we excluded certain studies, such as those that included exercise performed after revascularisation. However, this ensured that the review was manageable and as 64 trials were still included in spite of this, it is unlikely that including these studies would have altered our findings”. Many thanks for this suggestion.

**Comment:** Presentation of full data/ analysis of included studies. The authors might consider including a table summarising the findings for all studies. This is currently presented in the text but might be more informative/ easier for the reader to navigate in table form and collected according to study.

**Response:** Yes, we agree, and clarity is important. We have added the analysis and scoring of included studies in a table format. However, given this created seven new tables we have added these as supplementary materials (tables 3 and 4).

**Comment:** Minor inconsistency issues in use of SWT and ISWT abbreviations on pages 6 and 7.

**Response:** Many thanks for spotting this mistake, we have amended the manuscript accordingly.

**Comment:** Repetitions of reference in list – Hiatt et al 2005 is included twice (10 and 20). Perhaps review full list for any other inaccuracies

**Response:** This has now been rectified. Thankyou.

**Reviewer #2:**

**Comment:** I believe that RCTS of clinical trials or simply trial should also come in the title. And the authors should further justify why they choose only RCT trials since this is not an interventional review.

**Response:** Many thanks for this comment. We have now updated the title to “Review of Exercise Testing in Patients with Intermittent Claudication: A Focus on Test Standardisation and Reporting Quality in Randomised Controlled Trials”. We chose to exclude non-RCT’s in line with a previous review of reporting quality published in PLOS One (1). We also felt that this would ensure that the review was manageable and not overwhelming for the reader. Finally, standardised reporting is of the greatest importance in RCT’s as these often inform clinical practice or are used to generate level 1a evidence which does inform clinical practice. We have added the following sentence to the methods section “exclusion of these studies, and non-RCT's, is in line with a previous review on reporting standards and also ensured that the current review and the number of included studies was manageable”.

**Comment:** Next, in the inclusion criteria, the statement "studies that randomised patients to an exercise or comparator arm following revascularisation without a non-invasive exercise arm, were also excluded" is not quite clear. What does the authors mean by non-invasive exercise and of what relevance is it the review?

**Response:** We appreciate this comment and agree that this sentence is not as clear as it could be. To clarify, by non-invasive exercise arm, we meant an initial randomisation to exercise therapy or revascularisation, rather than post-revascularisation randomisation (i.e. randomised to revascularisation or exercise, rather than to exercise or control after revascularisation). We have edited this sentence to state “studies that randomised patients to an exercise or comparator arm following revascularisation were also excluded"

**Comment:** Furthermore, only full-text articles published from 1995 up to June 2020 were included, and the reason for this was based upon a meta analyses by Gardener and Poehlman, which has a different focus from this review. You need a better reason.

**Response:** We appreciate this comment, but our aim was to ensure that the review was manageable and not overwhelming to the reader. We felt that the 25-year period was sufficient especially given the increase in exercise-based trials in recent years. This is demonstrated in our results whereby we still included 64 studies. We have also added the following to the methods section “we excluded studies that were published prior to 1995 as the majority of exercise programmes after this date were based on a specific meta-analysis (14) and we felt that the period of 25 years would provide sufficient information to inform our findings, especially given the increase in exercise-based trials in recent years”.

**Comment:** The supplementary Table 1 is quite scanty and not in line with the focus of the review. Please upgrade this with more specific details.

**Response:** We have now added specific details regarding the exercise intervention and control (when available). Thankyou

We appreciate the opportunity to revise our manuscript and again wish to thank the reviewers and yourself.

Yours Sincerely,

Mr Sean Pymer – on behalf of all authors

**Reference:**

1.	Tew GA, Brabyn S, Cook L, Peckham E. The Completeness of Intervention Descriptions in Randomised Trials of Supervised Exercise Training in Peripheral Arterial Disease. PLOS ONE. 2016;11(3):e0150869.
